# An Internally Translated MAVS Variant Exposes Its Amino-terminal TRAF-Binding Motifs to Deregulate Interferon Induction

**DOI:** 10.1371/journal.ppat.1005060

**Published:** 2015-07-29

**Authors:** Arlet Minassian, Junjie Zhang, Shanping He, Jun Zhao, Ebrahim Zandi, Takeshi Saito, Chengyu Liang, Pinghui Feng

**Affiliations:** Department of Molecular Microbiology and Immunology, Norris Comprehensive Cancer Center, Keck School of Medicine, University of Southern California, Los Angeles, California, United States of America; Harvard Medical School, UNITED STATES

## Abstract

Activation of pattern recognition receptors and proper regulation of downstream signaling are crucial for host innate immune response. Upon infection, the NF-κB and interferon regulatory factors (IRF) are often simultaneously activated to defeat invading pathogens. Mechanisms concerning differential activation of NF-κB and IRF are not well understood. Here we report that a MAVS variant inhibits interferon (IFN) induction, while enabling NF-κB activation. Employing herpesviral proteins that selectively activate NF-κB signaling, we discovered that a MAVS variant of ~50 kDa, thus designated MAVS50, was produced from internal translation initiation. MAVS50 preferentially interacts with TRAF2 and TRAF6, and activates NF-κB. By contrast, MAVS50 inhibits the IRF activation and suppresses IFN induction. Biochemical analysis showed that MAVS50, exposing a degenerate TRAF-binding motif within its N-terminus, effectively competed with full-length MAVS for recruiting TRAF2 and TRAF6. Ablation of the TRAF-binding motif of MAVS50 impaired its inhibitory effect on IRF activation and IFN induction. These results collectively identify a new means by which signaling events is differentially regulated via exposing key internally embedded interaction motifs, implying a more ubiquitous regulatory role of truncated proteins arose from internal translation and other related mechanisms.

## Author Summary

Host innate immune signaling plays critical roles in defeating pathogen infection. In response to viral infection, cellular signaling events cumulate in the activation of NF-κB and interferon regulatory factors. How these two signaling ramifications are differentially regulated remains an open question. Here we report an internally translated MAVS variant deregulates IRF activation via exposing N-terminal TRAF-binding motifs. As such, the short form of MAVS efficiently competes for binding to TRAF2 and TRAF6 against full-length MAVS, thereby sequestering key adaptors from the signaling cascades mediated by full-length MAVS. Our study uncovers a delicate regulatory mechanism of truncated proteins bearing key protein-interacting motifs that is enabled by internal translation initiation and potentially other relevant means.

## Introduction

In response to pathogen infection, host cells initiate an immediate innate immune response to defeat pathogen propagation [1,2,3]. The retinoic acid-inducible gene I (RIG-I) and melanoma differentiation antigen 5 (MDA5) are cytosolic receptors that sense infecting viruses via RNA with distinct structural features [4,5,6]. Upon RNA association, RIG-I and MDA5 dimerize with the mitochondrion antiviral signaling (MAVS) adaptor that, in turn, triggers the activation of IKK (IKKα and β) and TBK-1 or IKKε (also known as IKKi) kinase [7,8,9,10]. IKKα or β phosphorylates the inhibitor of NF-κB and induces its subsequent degradation, unleashing NF-κB to translocate into the nucleus and up-regulate gene expression [11,12]. TBK-1 and IKKε phosphorylate the interferon regulatory factors (IRF) to enable the expression and secretion of interferons (IFN), e.g., interferon β [13,14]. As such, these signaling events cumulate in establishing an effective antiviral state.

To survive in the presence of active host immune defense response, pathogens have evolved a plethora of strategies to evade and exploit host immune signaling events [15,16]. RIG-I and MDA5 are key cytosolic sensors that detect intracellular RNA. Not surprisingly, RNA viruses deploy various mechanisms to disrupt signal transduction downstream of these receptors [16,17]. An elegant example that shared by multiple RNA viruses, including hepatitis C virus, picornavirus and enterovirus, is to cleave the MAVS adaptor off the mitochondrion membrane with a viral NS4/NS5 protease, thereby shutting down IFN induction in response to viral infection [9,18,19,20,21]. Our previous work with murine gamma herpesvirus 68 (γHV68), a model herpesvirus closely-related to Kaposi’s sarcoma-associated herpesvirus (KSHV) and Epstein-Barr virus (EBV), showed that γHV68 hijacks MAVS and IKKβ to promote viral transcriptional activation and disable cytokine gene expression [22,23,24]. As such, loss of MAVS impaired lytic replication of γHV68, in stark contrast to the increased viral replication of diverse RNA viruses [25,26]. These results suggest that gamma herpesviruses have evolved strategies to activate RIG-I and/or MDA5, receptors upstream of MAVS. Indeed, we have recently reported a new mechanism of RIG-I activation that is enabled by a viral pseudo enzyme [27]. These studies collectively defined intricate viral immune evasion and exploitation strategies.

The RIG-I and MDA5-dependent innate immune signaling bifurcates downstream of MAVS into the IKK-NF-κB and TBK-1/IKKε-IRF cascades. It is not well understood how these two signaling ramifications can be differentially regulated downstream of shared RIG-I and MAVS, and other common receptors and adaptors. We have recently discovered conserved gamma herpesvirus proteins, vGATs that preferentially activate NF-κB via deamidating RIG-I [27]. The mechanism of action of the herpesviral protein is unique in that: a) viral proteins, in contrast to RNA, activates RIG-I; b) these viral factors induce RIG-I activation via deamidation of key residues, a new means distinct from that induced by RNA; and c) the viral factor selectively triggers NF-κB activation, but not IRF activation and IFN induction, via RIG-I and MAVS. The differential activation downstream of MAVS by vGAT prompted us to dissect the underpinning mechanism. In search for factors that contribute to the differential activation of RIG-I-dependent signaling, we discovered a MAVS variant of ~50 kDa (designated MAVS50) that arises via internal translation from the second initiation codon. Notably, the MAVS50 variant was recently reported by Brubaker S.W. et al [28]. We discovered that, though lacking the N-terminal caspase recruitment domain (CARD), MAVS50 exposes its TRAF-binding motifs within the very N-terminus to deregulate IRF activation and IFN induction. Interestingly, MAVS50 is sufficient to activate NF-κB, but not IFN induction. MAVS50 inhibits MAVS70-mediated innate immune signaling via competing with full-length MAVS for binding to TRAF2 and TRAF6 molecules. Sum of both leads to the specific inhibition of IRF activation and IFN induction. Consequently, MAVS50 expression increased VSV lytic replication. Mutations ablating TRAF-binding ability impaired MAVS50 to inhibit IFN-β induction in response to Sendai virus (SeV) infection and to increase VSV replication. These findings identify a delicate mechanism of selective innate immune activation via truncated proteins that expose their key protein-interacting domains.

## Results

### Identification of MAVS50 during Innate Immune Activation

**Fig 1:**
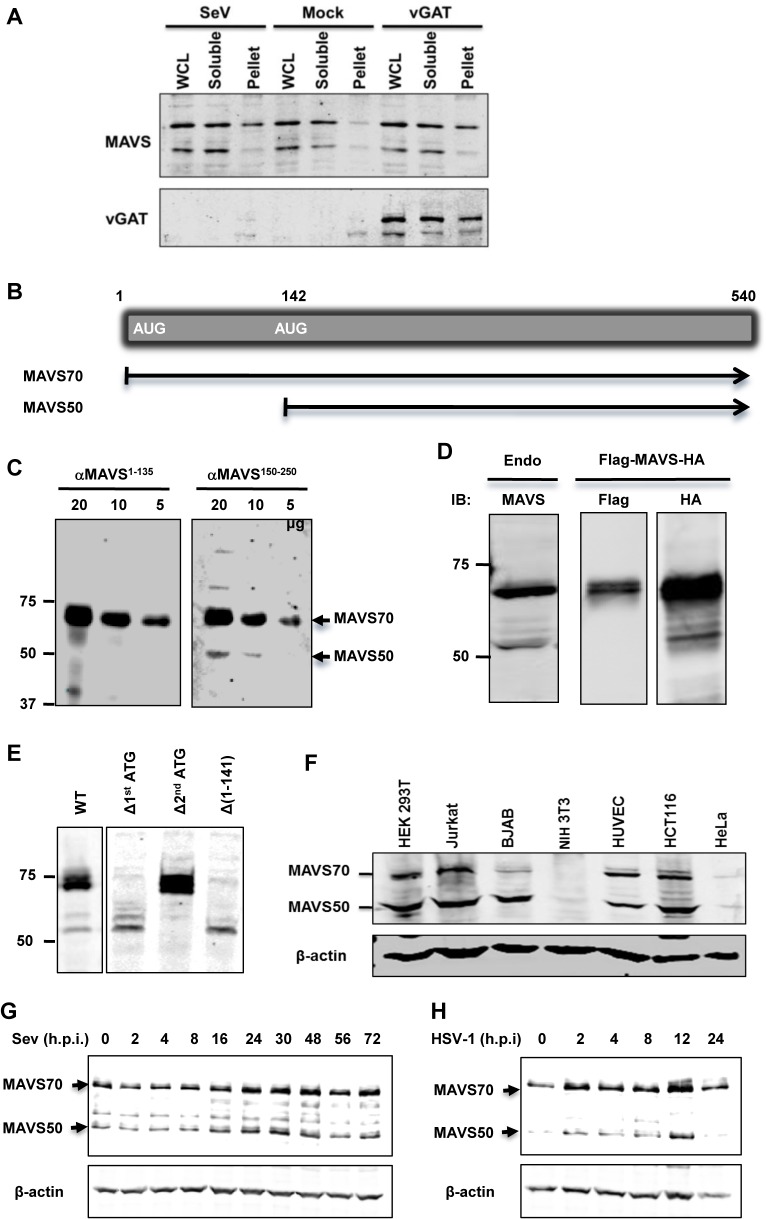
Identification of the MAVS50 variant. (A) 293T cells were infected with SeV (100 HA unit/ml) for 2 hours or transfected with a plasmid containing murine γHV68 vGAT for 24 hours. Whole cell lysates (WCL) were prepared in Triton X-100 buffer to separate into soluble and insoluble (pellet) fractions, which were analyzed by immunoblotting with indicated antibodies. (B) Diagram of the mRNA of the full-length MAVS (or MAVS70) and MAVS50. (C) WCLs of indicated amount were analyzed with antibodies against the first 135 amino acids (αMAVS^1-135^) or an internal sequence encompassing amino acids 150–250 (αMAVS^150-250^). (D) The expression of MAVS, carrying a N-terminal Flag tag and a C-terminal HA tag, in 293T cells was analyzed by immunoblotting with anti-Flag and anti-HA antibodies, along with endogenous MAVS (left panel). (E) 293T cells were transfected with plasmids containing wild-type MAVS and indicated mutants. WCLs were analyzed by immunoblotting with anti-MAVS antibody (αMAVS^150-250^). Δ, deletion. (F) WCLs of indicated cells were analyzed by immunoblotting with anti-MAVS (αMAVS^150-250^) and anti-β-actin. Note, antibody against human MAVS does not react with murine MAVS in NIH 3T3 cells. (G and H) 293T cells were infected with Sendai virus (SeV, 100 HA Unit/ml) (G) and HSV-1 (MOI = 5) (H) and cells were harvested at indicated time points. WCLs were analyzed by immunoblotting with antibody against MAVS.

We have previously identified gamma herpesviral homologues of glutamine amidotransferase (referred to as vGAT) activate RIG-I via deamidation [27]. We noted a remarkable feature of this innate immune activation is the preferential activation of the NF-κB signaling cascade, but not that of IRF and IFN induction. In an experiment that aims to examine MAVS activation by Sendai virus (SeV) infection or expression of γHV68 vGAT, we observed that a smaller isoform of MAVS, of ~50 kD (designated MAVS50), did not migrate into the Triton X-100-insoluble fraction in cells infected with SeV or expressing γHV68 vGAT (Fig 1A). In contrast, the full-length MAVS, designated as MAVS70, accumulated in the Triton X-insoluble fraction, indicative of its activation [8]. This result suggests that MAVS50 likely possesses function distinct from its kin, MAVS70.

We then set out to determine the nature of the MAVS50 variant. Visual inspection of MAVS mRNA revealed an internal translation initiation site at codon 142 (Fig 1B). We reasoned that MAVS50, if produced from internal translation from the second initiation codon, lacks the N-terminal region including the entire CARD domain. We employed two antibodies, one was raised against a polypeptide of the first 135 amino acids and the other against an internal sequence encompassing amino acids 150 to 250, to differentiate these two putative MAVS isoforms. When whole cell lysates were analyzed by immunoblotting, we found that only MAVS70 reacted with the antibody against the first 135 amino acids, whereas both MAVS70 and MAVS50 reacted with the antibody against the internal region (aa 150–250) (Fig 1C). This result indicates that MAVS50 lacks the amino-terminal region. We then engineered a MAVS construct that carries an amino-terminal Flag epitope and a carboxyl-terminal HA epitope to probe MAVS expression. Such a dually-tagged MAVS construct yielded a single MAVS species of 70 kDa reacting with anti-Flag antibody, and both species of ~70 kDa and 50 kDa reacting with anti-HA antibody. These two species are of similar sizes to endogenous MAVS (Fig 1D). To determine whether MAVS50 was produced from the second initiation codon, we mutated the second ATG (methionine) into TGC (cysteine) within the cDNA of MAVS70. To exclude that MAVS50 is a product of MAVS70 due to internal proteolytic cleavage, we deleted the first ATG of MAVS70. We also generated a construct that contains the cDNA sequence encoding amino acids 142 to 540 of MAVS70. As shown in Fig 1E, deletion of the first initiation codon abolished the expression of MAVS70, while produced more MAVS50 than a construct containing wild-type MAVS. The increased MAVS50 expression likely stems from the lack of competition of translation initiation at the first AUG codon. We noted additional species larger than 50 kDa were produced from the construct missing the first initiation codon, suggesting that these proteins are produced from internal translation using non-AUG codons upstream of the second AUG (142) initiation codon. As expected, mutation of the second initiation codon abolished the expression of MAVS50. Moreover, the MAVS construct containing the sequence encoding amino acids 142–540 yielded a MAVS protein migrating identically as MAVS50. These results collectively support the conclusion that MAVS50 is produced from internal translation initiation using the second AUG codon. Next, we probed a number of human cell lines for the expression of MAVS50 and found that MAVS50, similar to MAVS70, was abundantly expressed from HEK 293T, Jurkat T cells, BJAB B cells, HUVEC endothelial cells, HCT116 colorectal cells, although both isoforms were detected at very low level in HeLa cervical cells (Fig 1F). We further examined the expression of MAVS70 and MAVS50 expression in cells infected with SeV and herpes simplex virus type 1 (HSV-1), prototype RNA and DNA viruses, respectively. Both viruses modestly induced the expression of MAVS50 at late time points post-infection, specifically 24–48 (Fig 1G) and 12 (Fig 1H) hours post-infection for SeV and HSV-1, respectively.

### MAVS50 Activates NF-κB, but Not IRF and IFN Induction

**Fig 2:**
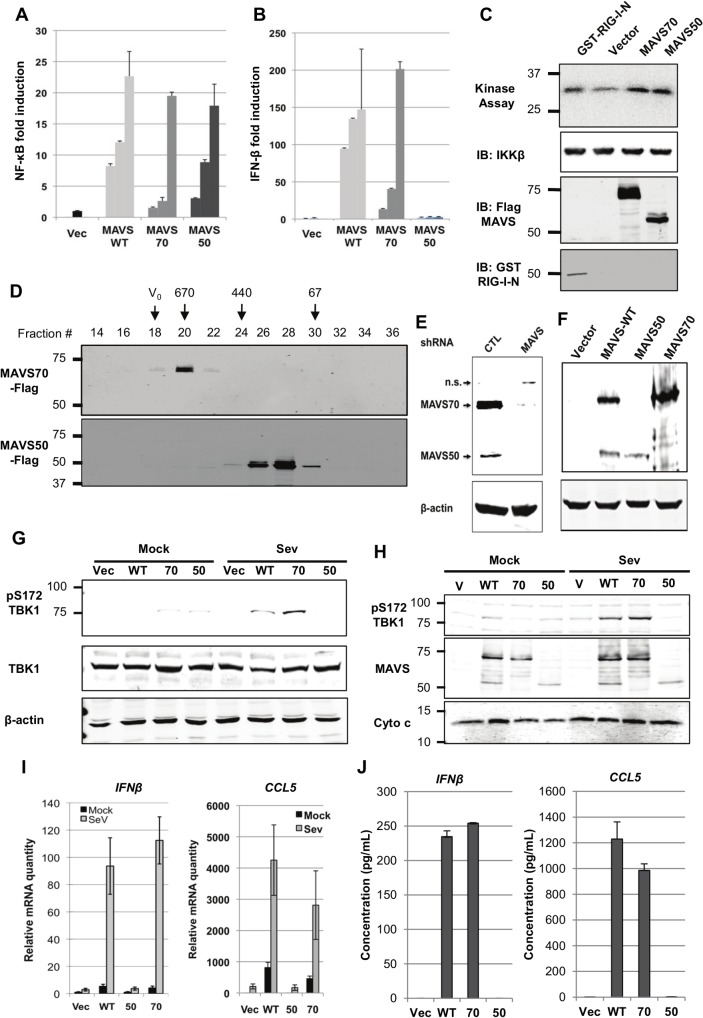
Characterize the roles of MAVS50 in RIG-I-dependent signaling. (A and B) 293T cells were transfected with an NF-κB (A) or IFN-β (B) reporter cocktail and increasing amount of MAVS wild-type (WT), MAVS70 or MAVS50. Reporter activation was determined by luciferase assay at 30 hours post-transfection. (C) 293T cells were transfected with plasmids containing indicated genes. At 48 hours post-transfection, IKKβ kinase was precipitated and analyzed by in vitro kinase assay and immunoblotting with anti-IKKβ. Whole cell lysates were analyzed with anti-Flag (MAVS) and anti-GST (RIG-I-N). (D) 293T cells were transfected with plasmids containing Flag-MAVS70 and Flag-MAVS50. MAVS70 and MAVS50 were purified by affinity chromatography, eluted and analyzed by gel filtration chromatography with Superdex 200. Fractions (30 μl) were analyzed by immunoblotting with anti-Flag antibody. V_0_, void volume; numbers at the top indicate molecular weight in kDa. (E and F) MAVS in 293T cells was depleted with shRNA and analyzed by immunoblotting (E) and MAVS expression was “reconstituted” with lentivirus containing MAVS wild-type (WT), MAVS70 or MAVS50. Whole cell lysates were analyzed with anti-V5 antibody (F). (G) MAVS knockdown 293T cells “reconstituted” with control lentivirus (Vec) or lentivirus containing MAVS wild-type (WT), MAVS70 (70) or MAVS50 (50) as shown in (F), were mock- or infected with Sendai virus (SeV, 100 HAU/ml) for 8 hours, WCLs were prepared and analyzed by immunoblotting with indicated antibodies. (H) Infection of “reconstituted” 293T cells as described in (G). Mitochondrion-enriched fraction was obtained and analyzed by immunoblotting with indicated antibodies. (I and J) MAVS knockdown 293T cells, “reconstituted” with MAVS expression as described in (F), were infected with SeV (100 HA unit/ml) for 8 hours, RNA was extracted, cDNA was prepared and real-time PCR with primers specific for *hIFNb* and *CCL5* were performed (I). Supernatants were collected and hIFNβ and hCCL5 were determined by ELISA (J).

MAVS serves as an adaptor to relay signaling from RIG-I and MDA5 receptor to downstream kinases that bifurcate to activate NF-κB and IRF transcription factors [7,8]. To examine the roles of MAVS50 in these signaling cascades, we over-expressed MAVS50 and examined signaling events leading to NF-κB and IRF activation. Using reporter assays, we found that MAVS50 expression activated NF-κB (Fig 2A) in a dose-dependent manner. By contrast, MAVS50 expression did not up-regulate the promoter of IFN-β, but MAVS wild-type and MAVS70 did (Fig 2B). Consistent with the NF-κB activation, over-expressed MAVS50 also up-regulated the kinase activity of IKKβ by an in vitro kinase assay, in comparison to RIG-I-N and MAVS70 (Fig 2C). TRAF molecules are important adaptors downstream MAVS and are implicated in specific activation of NF-κB and IRF transcription factors. Thus, we examined MAVS50 interactions with a panel of six TRAF molecules by co-IP assays in transfected 293T cells. While MAVS70 interacted with all TRAF molecules except TRAF4, MAVS50 demonstrated preferential interaction with TRAF2 and TRAF6 (S1A and S1B Fig). This result suggests that MAVS50, in comparison to MAVS70, is distinct in interacting with downstream TRAF adaptors. When signaling events of TBK-1 and IRF activation were examined, we found that MAVS50 expression had no detectable effect on the kinase activity of TBK-1, nor the dimerization of IRF3 (S1C and S1D Fig), supporting the conclusion that MAVS50 does not activate the IRF signaling cascades. As controls, RIG-I-N (2CARDs) and MAVS70 potently activated TBK-1 by kinase assay and induced IRF3 dimerization by native gel electrophoresis. These results collectively show that MAVS50 preferentially activates the IKKβ-NF-κB signaling cascade.

MAVS is characterized by a CARD-mediated oligomerization in provoking downstream signaling events [8,29]. MAVS50 lacks the CARD domain and we determined whether MAVS50 forms oligomers by size exclusion chromatography. When purified from 293T cells, MAVS70 was eluted in fractions corresponding to ~670 kDa, consistent with the notion that MAVS70 forms large oligomers (Fig 2D). However, MAVS50 was eluted in fractions corresponding to ~120 kDa, suggesting that MAVS50 forms smaller oligomers, likely dimer or tetramer. This result is consistent with the critical roles of CARD domain in mediating large signaling-competent oligomers [30,31]. Given that MAVS50 lacks the CARD for interaction with RIG-I, we determined whether MAVS50 could relay signal transduction downstream of RIG-I. Thus, we knocked down the expression of endogenous MAVS (both isoforms) (Fig 2E) and “reconstituted” MAVS expression with MAVS wild-type, MAVS70 or MAVS50 (Fig 2F). These “reconstituted” cells were then used to examine RIG-I-dependent signaling in response to SeV infection. Upon SeV infection, 293T cells “reconstituted” with wild-type MAVS and MAVS70 activated TBK-1 as determined by TBK-1 phosphorylation at ser172, a marker for TBK-1 activation (Fig 2G). However, MAVS50 expression failed to do so. When mitochondrion-enriched fractions were analyzed for phosphorylated TBK-1 (pS172), we found that phosphorylated TBK-1 was abundant in the mitochondrion-enriched fraction from cells “reconstituted” with wild-type MAVS and MAVS70, but not that of cells expressing MAVS50 (Fig 2H). Consistent with this, “reconstituted” expression of wild-type MAVS and MAVS70 restored robust expression of *IFNb*, *CCL5*, *ISG56*, *IL-8* and *Viperin*, indicative of activation of IRF and NF-κB (Fig 2I and S2A Fig). However, “reconstituted” expression of MAVS50 failed to up-regulate the transcription of these anti-viral cytokines. Enzyme-linked immunoassay (ELISA) further show that IFN and CCL5 were produced from knockdown cells expressing wild-type MAVS and MAVS70 (Fig 2J). We observed marginal but detectable level of *IFNb* and significantly *ISG56* in resting cells that were “reconstituted” for the expression of MAVS wild-type and MAVS70. Similar to MAVS wild-type and MAVS70, MAVS50 localizes to the mitochondrion and peroxisome when expressed in 293T cells (S2B and S2C Fig), consistent with the notion that MAVS is targeted to the mitochondrion via a transmembrane tail [8]. Thus, MAVS50 does not relay signal transduction from RIG-I to downstream molecules, re-enforcing the critical role of CARD in RIG-I-mediated signaling.

### MAVS50 Interacts with MAVS70

**Fig 3:**
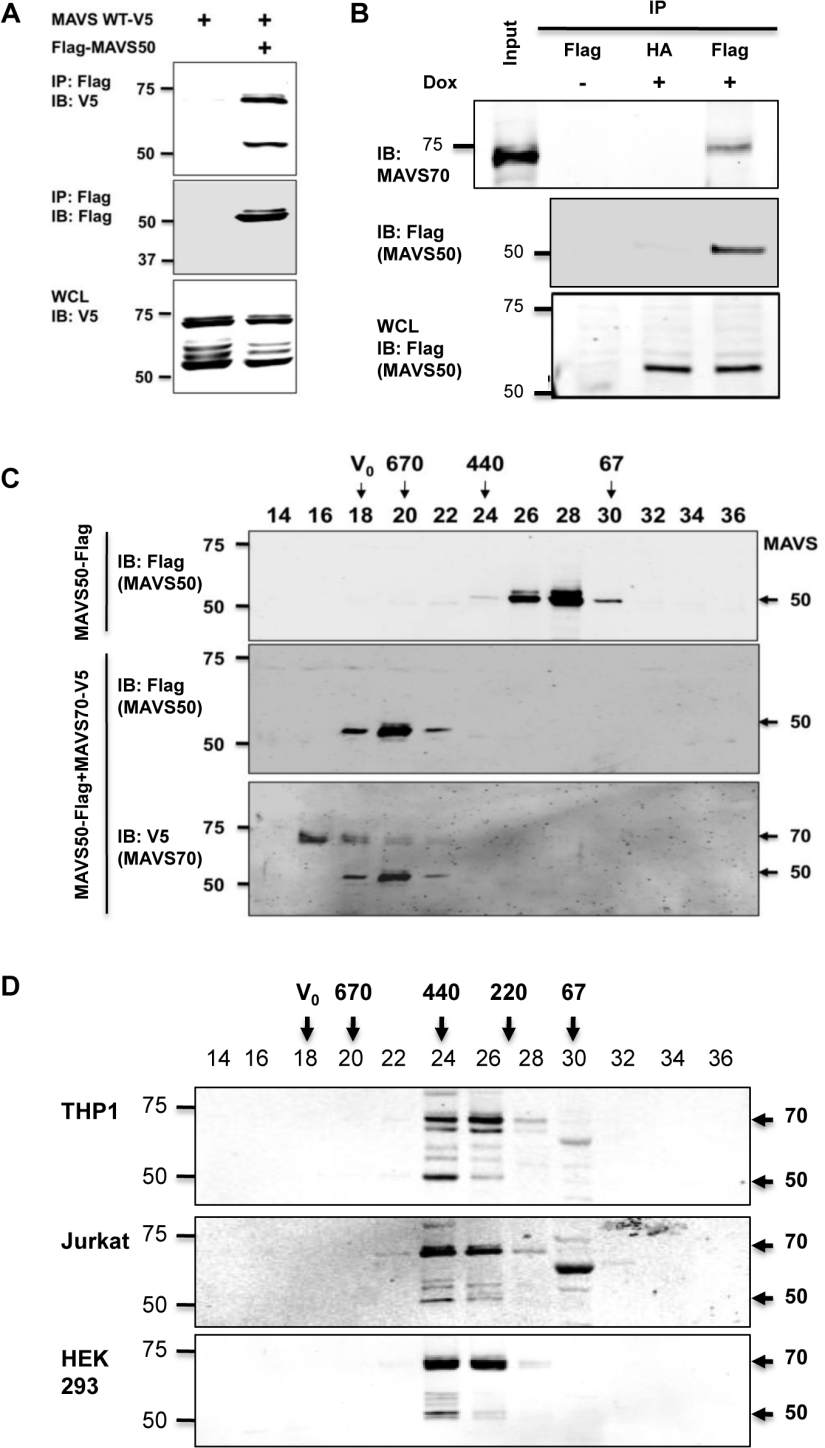
MAVS50 interacts with MAVS70. (A) 293T cells were transfected with plasmids containing MAVS wild-type, MAVS70 or MAVS50. Whole cell lysates (WCLs) were precipitated with anti-Flag (MAVS50). Precipitated proteins and WCLs were analyzed by immunoblotting with indicated antibodies. (B) 293T-Rex/MAVS50-Flag cell line was induced with doxycycline (100 ng/ml) for 24 hours. WCLs were precipitated with anti-Flag agarose (MAVS50). Precipitated proteins and WCLs were analyzed by immunoblotting with indicated antibodies. (C) 293T cells were transfected with a plasmid containing MAVS50-Flag, with or without a plasmid containing MAVS70-V5. MAVS50 was purified by affinity chromatography, eluted and analyzed by gel filtration chromatography. Fractions (30 μl) were analyzed by immunoblotting with anti-Flag and anti-V5 antibodies. (D) Whole cell lysates of 293T, HeLa and THP-1 macrophage were analyzed by gel filtration chromatography. Fractions (50 μl) were analyzed by immunoblotting with anti-MAVS antibody. For C and D, V_0_, void volume; numbers indicate molecule weight in kDa.

MAVS50 lacks the CARD domain and fails to relay signal transduction downstream of RIG-I. Considering that MAVS50 possesses most of the sequence of MAVS70, we reasoned that MAVS50 likely regulates MAVS70-mediated signaling. To test this hypothesis, we first examined whether MAVS50 can interact with MAVS70 and itself. In transfected 293T cells, V5-tagged MAVS70 and MAVS50 were readily detected in protein complexes precipitated with anti-Flag antibody against Flag-MAVS50 (Fig 3A), indicating that MAVS50 can interact with MAVS70 and MAVS50. This result is consistent with our observation that MAVS50 eluted as ~120 kDa in gel filtration, which implies self-oligomerization of MAVS50 (Fig 2D). Due to the largely overlapping sequence between MAVS70 and MAVS50, it is technically challenging to probe the interaction between endogenous MAVS70 and MAVS50. Thus, we established a stable 293 cell line that expresses Flag-tagged MAVS50 under the control of doxycycline in a dose-dependent manner (S2D Fig). Precipitation of MAVS50 effectively pulled down MAVS70, indicating that MAVS50 physically associates with endogenous MAVS70 (Fig 3B).

We further analyzed the interaction between MAVS70 and MAVS50 with gel filtration that is routinely used to assess protein complex formation. Purified MAVS50 was predominantly eluted in fractions corresponding to proteins of ~120 kDa. However, when MAVS50 and MAVS70 were co-expressed in 293T cells, purified MAVS50 was eluted in fractions corresponding to ~670 kDa oligomer (Fig 3C). These results show that MAVS70 can convert MAVS50 into oligomers of larger sizes. Moreover, MAVS70 was also detected in fractions that were enriched with oligomerized MAVS50, indicating that MAVS70 is integrated in the MAVS50 oligomers and vice versa (Fig 3C). Finally, we assessed the elution pattern of MAVS70 and MAVS50 in lysates of three representative cell lines, including THP-1 monocyte, 293T fibroblast and HeLa cervical epithelial cells, by size exclusion chromatography. MAVS70 and MAVS50 co-eluted in fractions corresponding to ~220–440 kDa in THP-1 monocytes, 293T and HeLa cells (Fig 3D). A notable difference in the elution patterns of MAVS70 and MAVS50 was observed, i.e., MAVS70 were more evenly distributed in fractions 24 and 26, whereas MAVS50 was predominantly eluted in fraction 24. Furthermore, upon Sendai virus infection, MAVS50 increased in fraction 26 that MAVS70 peaked in elution (S3 Fig). Similar result was observed for 293T cells infected with murine gamma herpesvirus 68, a DNA virus (S3 Fig). These observations suggest that MAVS 50 preferentially associate with the larger size of MAVS70 oligomers. Collectively, these results indicate that MAVS50 physically interacts with MAVS70.

### MAVS50 Inhibits IFN-β Induction

**Fig 4:**
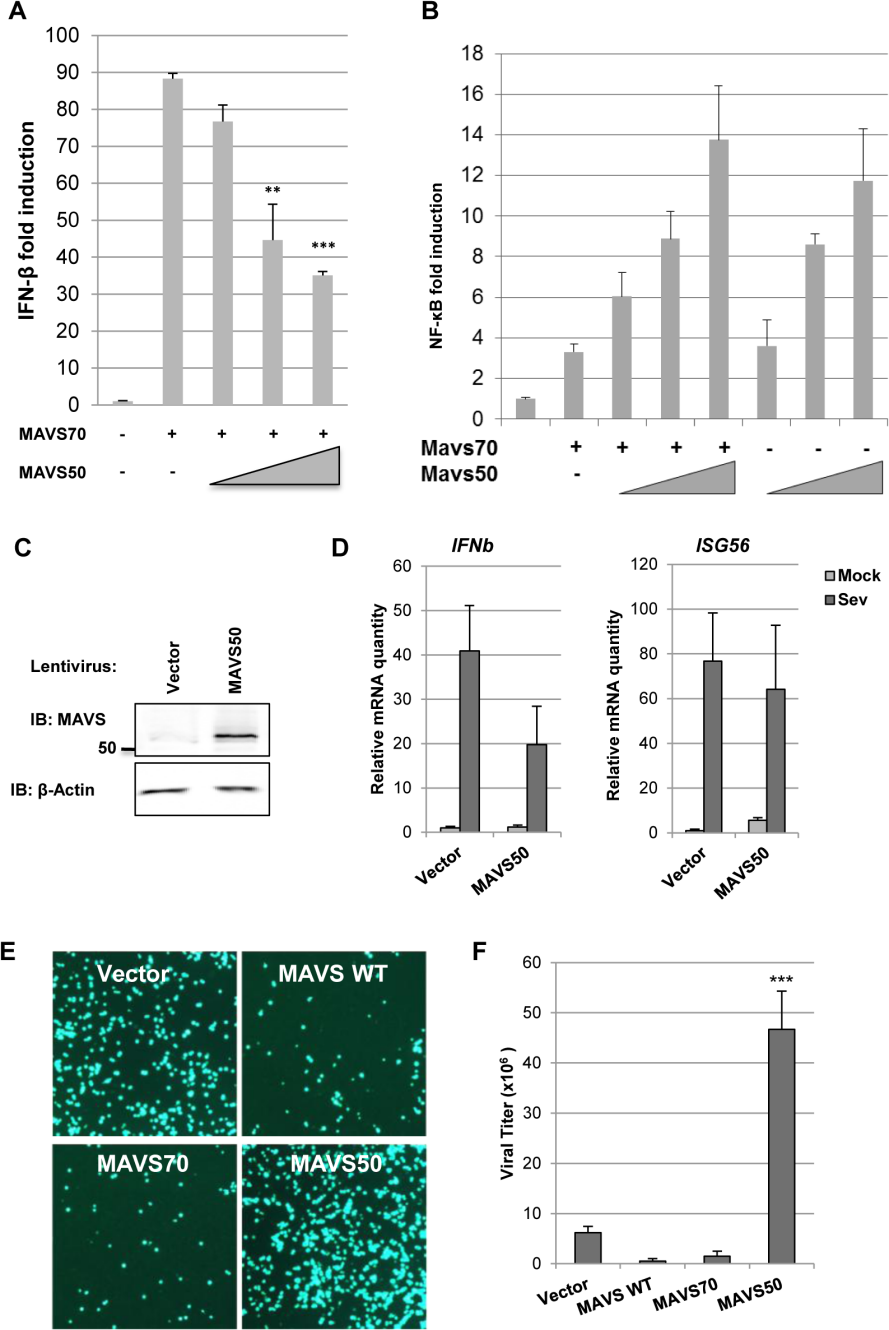
MAVS50 inhibits MAVS70-dependent IFN induction. (A and B) 293T cells were transfected with an IFN-β (A) or NF-κB reporter cocktail (B), a plasmid containing MAVS70 and increasing amount of a plasmid containing MAVS50. The promoter activity of IFN-β and NF-κB was determined by luciferase assay at 30 hours post-transfection. **p<0.*01*; ***p<0.*005*. (C and D) 293T cells were infected with control (CTL) lentivirus or lentivirus containing MAVS50. Whole cell lysates were analyzed with indicated antibodies (C). Stable 293T cells were infected with SeV (100 HA unit/ml) for 8 hours and RNA was extracted. cDNA was prepared and analyzed by real-time PCR with primers specific for *IFNβ* and *ISG56* (D). (E and F) 293T cells were transfected with vector or plasmids containing MAVS wild-type (WT), MAVS70 or MAVS50. At 24 hours post-transfection, cells were infected with VSV-GFP (MOI = 0.01). Cells were photographed at 24 hours post-infection (E) and VSV in the supernatant was determined by plaque assay (F).

To determine the effect of MAVS50 on MAVS70-mediated signaling, we assessed activation of NF-κB and IFN-β promoter by reporter assays. We found that MAVS50 inhibited MAVS70-induced transcription of the IFN-β promoter in a dose-dependent manner (Fig 4A). By contrast, MAVS50 did not significantly impact the NF-κB activation by MAVS70 (Fig 4B). We noted that MAVS50 was capable of activating NF-κB. We then expressed exogenous MAVS50 either by lentivirus transduction or transient transfection, and examined host cytokine gene expression and viral replication. When MAVS50 was expressed in 293T cells by lentivirus transduction (Fig 4C), while *ISG56* expression was not significantly impacted, the expression of *IFNβ* was reduced by 50% in response to SeV infection (Fig 4D). Conversely, exogenously expressed MAVS50 enhanced the replication of vesicular stomatitis virus (VSV), a prototype RNA virus, by fluorescence microscopy (Fig 4E). Plaque assay further showed that MAVS50 expression increased VSV replication by more than 5-fold (Fig 4F). Taken together, MAVS50 inhibits IFN-β induction in response to viral infection.

### MAVS50 Competes with MAVS70 for Binding to TRAF Molecules

**Fig 5:**
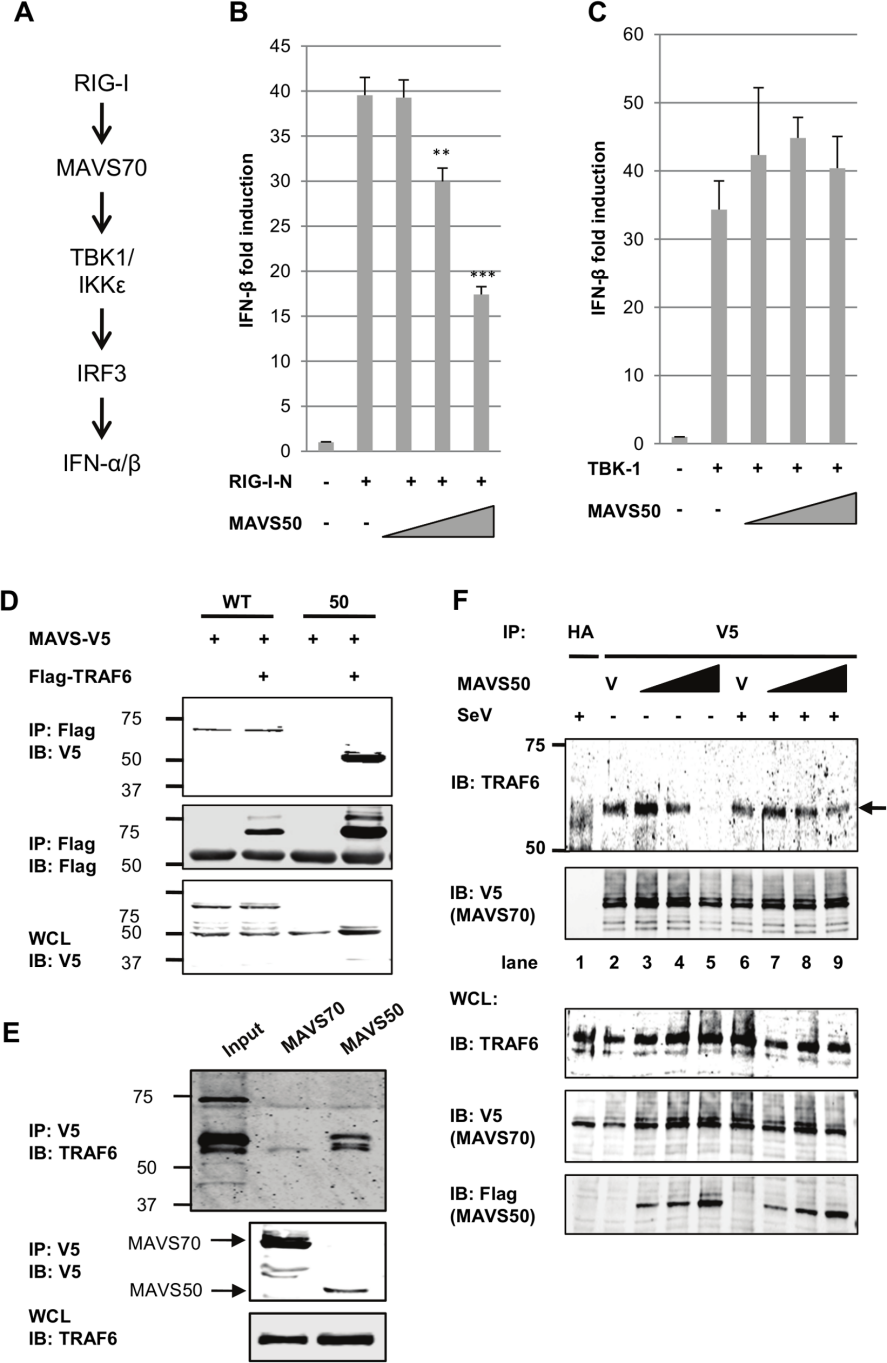
MAVS50 targets TRAF molecules to inhibit MAVS70-mediated IFN induction. (A) Diagram of key signaling molecules of the RIG-I-dependent IFN induction. (B and C) 293T cells were transfected with an IFN-β reporter cocktail, a plasmid containing RIG-I-N (B) or TBK-1 (C), and increasing amount of plasmid containing MAVS50. The IFN-β promoter activity was determined by luciferase assay at 30 hours post-transfection. **p<0.*01*; ***p<0.*005*. (D) 293T cells were transfected with plasmids containing MAVS wild-type (WT) or MAVS50 and a plasmid containing TRAF6. Whole cell lysates (WCL) were precipitated with anti-Flag (TRAF6). Precipitated proteins and WCLs were analyzed by immunoblotting with indicated antibodies. (E) 293T cells, depleted with endogenous MAVS and “reconstituted” with MAVS70 or MAVS50, were precipitated with anti-V5 (MAVS70 or MAVS50). Precipitated proteins and WCLs were analyzed by immunoblotting with indicated antibodies. (F) MAVS knockdown cells “reconstituted” with MAVS70-V5 were transfected with increasing amount of MAVS50-Flag. At 30 hours post-transfection, cells were infected with Sendai virus (SeV, 200 HA unit/ml) for two hours. WCLs were prepared and precipitated with anti-V5 (MAVS70) antibody. Precipitated proteins and WCLs were analyzed by immunoblotting with indicated antibodies. V, vector.

The cytosolic sensor-mediated IFN induction pathway constitutes of key signaling molecules, including RIG-I/MDA5, MAVS, TBK-1/IKKε, and IRF3 (Fig 5A). Over-expression of these components is sufficient to activate downstream signaling events, cumulating in the up-regulation of IFN expression. To identify the point of inhibition by MAVS50, we employed reporter assay that takes advantage of the IFN-β promoter as a surrogate and the over-expressed key components outlined in Fig 5A. While MAVS50 inhibited transcription of the IFN-β promoter induced by RIG-I-N and MAVS70 (Figs 5B and 3D), MAVS50 had no inhibitory effect on the IFN-β promoter induced by TBK-1, IKKε and the constitutively active IRF3-5D mutant (Fig 5C and S4A and S4B Fig). These results suggest that MAVS50 targets a step between MAVS70 and TBK-1, a link between the common adaptor and bifurcated downstream signaling events that trigger IRF activation and IFN induction.

Considering that MAVS50 contains TRAF2- and TRAF6-binding motifs within its N-terminus and that TRAF molecules serve as link downstream of MAVS, we reasoned that MAVS50 likely targets TRAF molecules to modulate MAVS70-mediated signaling of the IRF branch. We then assessed TRAF6 interaction with MAVS70 and MAVS50 by co-immunoprecipitation. When TRAF6 was precipitated in transfected 293T cells, MAVS50 was readily detected and MAVS70 was detected at background level (Fig 5D). We noted that MAVS70 expression consistently diminished the interaction between MAVS50 and TRAF6. We further probed TRAF6 interaction with MAVS50 or MAVS70 in 293T cells that were “reconstituted” with exogenous MAVS70 and MAVS50. Co-IP assays demonstrated that TRAF6 was readily detected when MAVS50 was precipitated in 293T cells (Fig 5E). Although TRAF6 was detected at basal level when co-precipitated with MAVS70, we believe this is likely due to the strong interaction between MAVS50 and TRAF6. Interestingly, although MAVS50 can interact with TRAF3, MAVS50 failed to precipitate with TRAF3 in the presence of MAVS70 (S4C Fig). This result suggests that MAVS50 weakly interacts with TRAF3.

To determine whether MAVS50 can compete with MAVS70 for binding to TRAF6, we took advantage of the MAVS knockdown cells that were “reconstituted” with exogenous V5-tagged MAVS70 to examine MAVS70 interaction with TRAF6 by Co-IP assay. We found that increasing amount of MAVS50 reduced TRAF6 precipitated with anti-V5 (MAVS70) in a dose-dependent manner (Fig 5F). Interestingly, MAVS50 expressed at a lower level increased the amount of TRAF6 precipitated with MAVS70 (compare lane 2 and 3), suggesting that MAVS50 integrates into MAVS70 to bridge an interaction between MAVS70 and TRAF6. With higher levels of MAVS50, MAVS50 effectively reduced TRAF6 precipitated by MAVS70, indicative of competition between MAVS50 and MAVS70 for association with TRAF6 (compare lanes 4 and 5 to lanes 2 and 3). In SeV-infected cells, the effect of MAVS50 on interaction between MAVS70 and TRAF6 was reduced, suggesting that SeV infection partly inhibits MAVS50 action. We also recognized that SeV infection slightly reduced, rather than increased, MAVS70 interaction with TRAF6. This is likely due to the “reconstituted” expression of MAVS70 that already activated downstream signaling in resting cells (Fig 2I) and further activation by SeV infection likely precipitated MAVS70 out from the Triton X-100-soluble fraction. Nevertheless, MAVS50 competes with MAVS70 for binding to TRAF6.

### A N-Terminal TRAF2-Binding Motif Is Crucial for MAVS50 to Interact with TRAF Molecules and Inhibit IRF Activation

**Fig 6:**
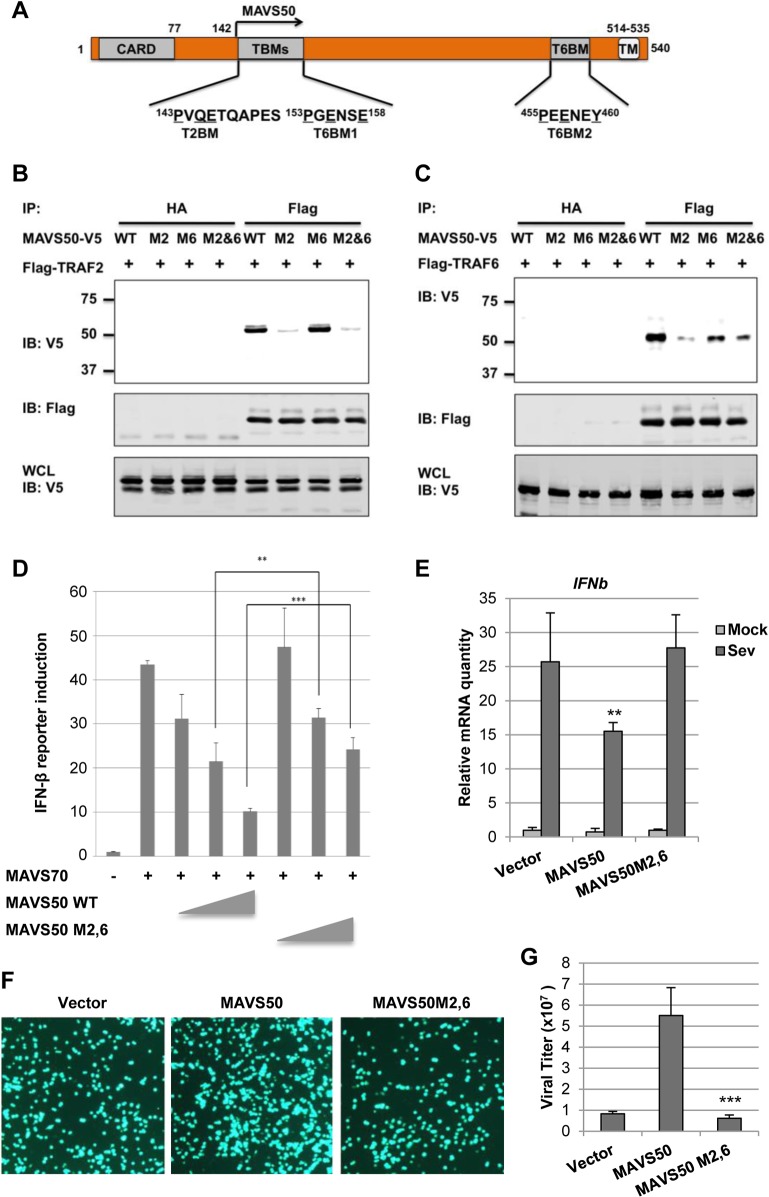
The N-terminal TRAF2-binding motif is critical for MAVS50 to inhibit IFN induction. (A) Diagram of the TRAF2-binding motif (T2BM) and TRAF6-binding motif (T6BM) in MAVS50, in relation to MAVS70. (B and C) 293T cells were transfected with plasmids containing Flag-TRAF2 (B) or Flag-TRAF6 (C) and plasmids containing MAVS50 wild-type (WT), mutant of TRAF2-binding (M2) or TRAF6-binding (M6) or both TRAF2- and TRAF6-binding (M2,6). Whole cell lysates (WCLs) were prepared at 30 hours post-transfection and precipitated with anti-Flag agarose or anti-HA agarose (as negative control). Precipitated proteins and WCLs were analyzed by immunoblotting with indicated antibodies. (D) 293T cells were transfected with an IFN-β reporter cocktail, a plasmid containing MAVS70 and increasing amount of a plasmid containing MAVS50 wild-type (WT) or MAVS50 M2,6 mutant. The IFN-β promoter activity was determined by luciferase assay at 30 hours post-transfection. **p<0.*01*; ***p<0.*005*. (E) 293T cells were infected with control lentivirus (CTL) or lentivirus containing MAVS50 wild-type or MAVS50 M2,6 mutant. At 48 hours, cells were infected with SeV (100 HA unit/ml) for 8 hours. RNA was extracted and cDNA were prepared for real-time PCR analysis with primers specific for *IFNβ* and *ISG56*. (F and G) 293T cells were transfected with an empty plasmid (Vector) or a plasmid containing MAVS50 or MAVS50 M2,6 mutant. At 24 hours post-transfection, cells were infected with VSV-GFP (MOI = 0.01). Cells were photographed at 24 hours post-infection (F) and virus in the supernatant was determined by plaque assay (G).

A major TRAF2-binding motif (PVQE, [P/S/A/T]x[Q/E]E) locates at the very amino terminus of MAVS50 and a TRAF6-binding motif (PGENSE, PxExx[Ar/Ac]; Ar, aromatic; Ac, acetic)[32,33] immediately follows the TRAF2-binding motif (Fig 6A). To probe the contribution of these N-terminal TRAF-binding motifs, we mutated the critical residues of TRAF2- and TRAF6-binding motifs into alanines, thus named M2 and M6 of MAVS50 (S5A Fig), and examined their interactions with TRAF2 or TRAF6 by co-IP assay. As expected, mutations within the TRAF2-binding motif abolished MAVS50 interaction with TRAF2, while mutations within the TRAF6-binding motif had no effect on MAVS50 interaction with TRAF2 (Fig 6B). MAVS50 mutant ablated both TRAF2- and TRAF6-binding motifs lost the interaction with TRAF2. Surprisingly, mutating the TRAF2-binding motif nearly abolished the MAVS50 interaction with TRAF6 (Fig 6C). Mutations within the immediate downstream TRAF6-binding motif, although reduced MAVS50 interaction with TRAF6, had less effect than mutations within the TRAF2-binding motif (Fig 6C). Simultaneously mutating the TRAF2- and TRAF6-binding motifs reduced MAVS50 association with TRAF6 to residual level. The residual level of TRAF6 interaction of MAVS50 M2,6 mutant is likely due to the transmembrane-proximal TRAF6-binding motif. Additionally, the MAVS50 M2,6 mutant also demonstrated reduced interactions with TRAF3 and TRAF5 in transfected 293T cells (S5B and S5C Fig). Mutations within the TRAF2- and TRAF6-binding motifs had no significant effect on MAVS50 interaction with MAVS70 by co-IP assay (S5D Fig), suggesting that these MAVS50 mutants are functionally competent. These results indicate that the very amino-terminal TRAF2-binding motif is critical for binding to both TRAF2 and TRAF6, and likely other TRAFs, suggesting that the TRAF2-binding motif is a functionally degenerate interaction motif for more than one TRAF molecule.

To determine the role of the N-terminal TRAF-binding motifs of MAVS50, we examined the effect of MAVS50 mutant that harbors mutations in both TRAF2- and TRAF6-binding motifs, designated MAVS50-M2,6. Compared to wild-type MAVS50, MAVS50-M2,6 was significantly impaired to inhibit MAVS70-induced IFN-β expression by reporter assay (Fig 6D). We then expressed exogenous MAVS50 or MAVS50M2,6 mutant by lentivirus (S5E Fig) and infected these cells with SeV. Real-time PCR analysis showed that the MAVS50-M2,6 mutant failed to inhibit *IFNb* gene expression in response to SeV infection, but wild-type MAVS50 did (Fig 6E). Consistent with this, wild-type MAVS50 expression increased VSV replication, but MAVS50-M2,6 mutant completely lost the ability to promote VSV replication (Fig 6F and 6G). These results collectively demonstrate the critical role of the TRAF-binding motifs of MAVS50, locating within the very N-terminal region, in inhibiting IFN induction downstream of RIG-I.

## Discussion

Upon sensing pathogen-associated molecular patterns, pattern recognition receptors initiate signaling events that bifurcate into NF-κB and IRF activation downstream of common adaptor molecules. While NF-κB activation and cytokine production are important for inflammatory response that attracts other immune cells to the site of infection, activated IRF and secreted IFN exert immediate antiviral effect within the site of infection. How NF-κB and IRF activation, triggered by shared upstream signaling molecules such as RIG-I and MAVS, are differentially regulated is not well understood. We report here that the MAVS50 variant, translated from an internal initiation codon, effectively competes with MAVS70 for binding to TRAF molecules. In doing so, MAVS50 inhibits MAVS70-mediated signal transduction, specifically IRF activation and interferon induction. Our work agrees well with a recent study reporting that MAVS50 suppressed RIG-I-dependent IFN induction [28]. Additionally, linear ubiquitination of NEMO, the scaffold protein of IKK and TBK-1 kinase complexes, was previously reported to dampen IFN induction while stimulating NF-κB activation [34]. This activity requires the LUBAC E3 ligase that catalyzes linear ubiquitin chain assembly on NEMO and disrupts MAVS interaction with TRAF3, which relays signaling from MAVS to TBK-1 and IRF activation. A more recent study showed that cholera toxin induced RIG-I-dependent signaling events with a signature of NF-κB activation, although the molecular mechanism underpinning this preferential activation is not clear [35]. These studies, including our current work, highlight a recurring theme in differential regulation of the two signaling ramifications downstream of shared receptors that sense invading pathogens, pointing to a common shift from IFN induction to a NF-κB-dependent inflammatory response. Under conditions of pathogen infection, it is likely that IFN induction is the immediate robust response of the innate immune phase, whereas NF-κB activation and cytokine secretion constitute a modest but sustained inflammatory response. Given the pro-survival roles of NF-B activation, it is not surprising that viruses often usurp NF-κB activation or upstream signaling events to facilitate their infection, such as HIV and herpesviruses [15,36]. By contrast, IRF activation and IFN signaling promote cell death. In contrast to what was reported by Brubaker et al., we did not observe cell death induced by MAVS70 and MAVS50. This discrepancy may stem from the difference in our experimental conditions.

Despite of missing the N-terminal CARD domain that mediates hetero-oligomerization with RIG-I and self-oligomerization of MAVS [30,31], MAVS50 forms small oligomers that correspond to the size of a dimer or tetramer analyzed by gel filtration. Consistent with that, MAVS50 can homo-dimerize as determined by co-immunoprecipitation. This intriguing observation suggests the existence of unknown sequence that, in addition to CARD, mediates MAVS dimerization. To define a dimerization domain, we have applied serial truncations from the N-terminus of MAVS50 and performed co-IP assays. Unfortunately, we failed to pinpoint a key homo-dimerization sequence, implying that MAVS50 homo-dimerization requires a structural sequence, rather than the primary linear sequence. Alternatively, other cellular factors, including mitochondrial membrane, may scaffold the homo- and hetero-dimerization of MAVS70 and MAVS50. Nevertheless, MAVS50 is prone to form oligomer and, when it is over-expressed, is sufficient to trigger NF-κB activation. On the other hand, “reconstituted” expression of MAVS50 in cells that endogenous MAVS isoforms, both MAVS70 and MAVS50, were depleted by shRNA-mediated knockdown failed to trigger the expression of inflammatory cytokines and IFN-β in response to SeV infection. This result indicates that MAVS50 can not relay signal transduction from RIG-I to NF-κB and IRF transcription factors and requires MAVS70 to do so, re-enforcing the critical role of the CARD domain in assembling the RIG-I-MAVS signaling platform. Indeed, MAVS50 interacts with MAVS70 and MAVS70 expression converted MAVS50 from oligomers of ~120 to those of ~670 kDa. When purified MAVS50 was analyzed by gel filtration, MAVS70 was detected in fractions that were enriched for oligomerized MAVS50. Thus, MAVS70 can incorporate into MAVS50 oligomers, and vice versa. These activities enable MAVS50 to serve as a modulator of the MAVS-dependent immune pathways via interaction with MAVS70 and TRAF molecules, key amplifiers at the crossroad in innate immune signaling. However, MAVS70 expression diminished the interaction between MAVS50 and TRAF molecules, including TRAF6 and TRAF3. These results suggest that the innate immune signaling, activated by MAVS70 expression, is capable of inactivating MAVS50 and potentially releasing the MAVS50-mediated inhibition. This hypothesis remains to be examined in the near future.

**Fig 7:**
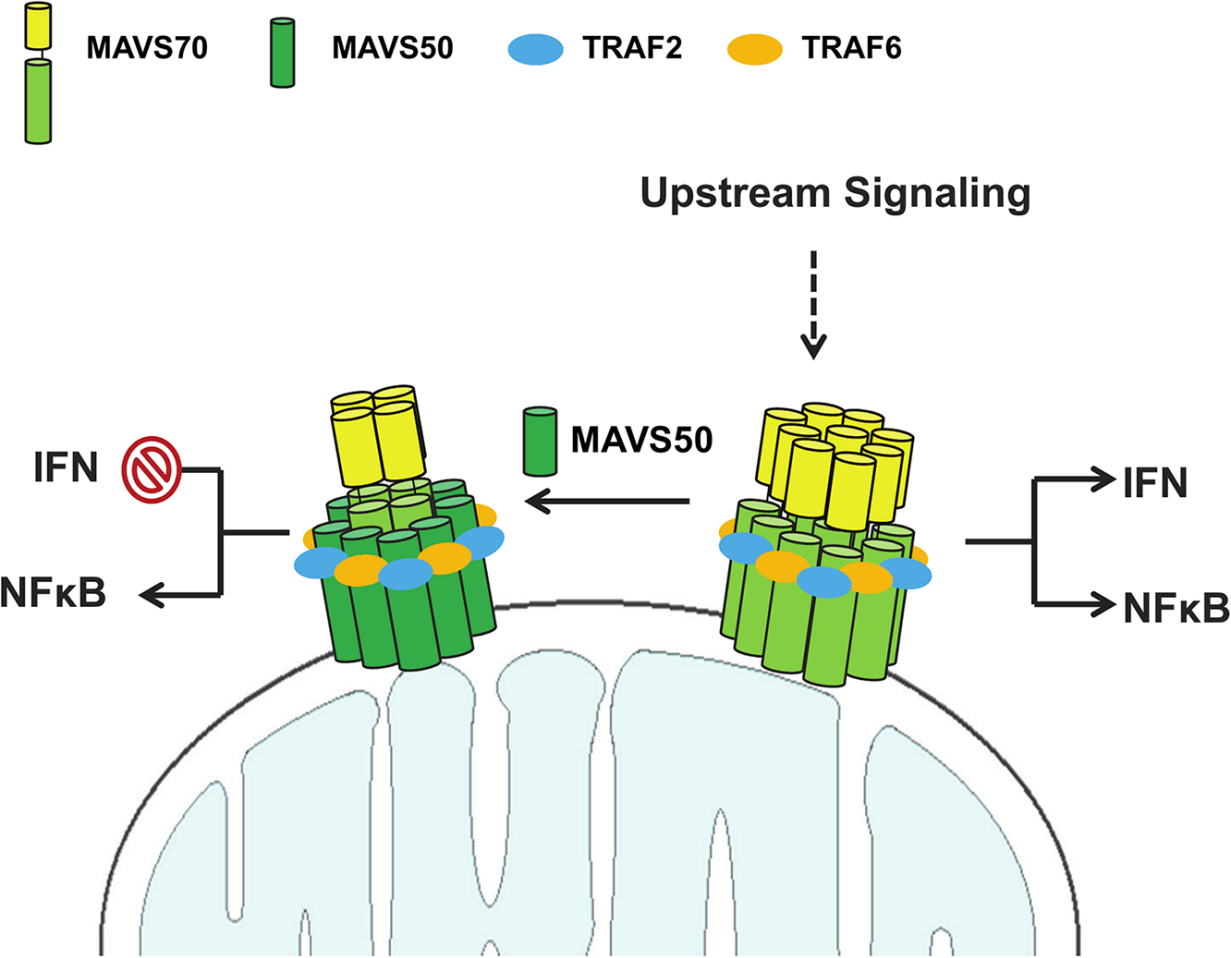
A hypothetical model on MAVS50 in modulating MAVS70-dependent signaling. Upon receiving upstream activation signal, MAVS70 forms large oligomer that triggers both NF-κB activation and IRF activation and IFN induction. MAVS50 interacts with MAVS70 and integrates into the MAVS70 oligomers. MAVS50 recruits significant portion of TRAF6 and TRAF2 with its N-terminally exposed TRAF-binding motifs, resulting in the inhibition of MAVS70-dependent NF-κB and IRF activation. The oligomer containing MAVS70 and MAVS50 activates only NF-κB, but not IRF-IFN branch. As such, MAVS50 selectively inhibits IFN induction. See discussion section for more detail.

How does MAVS50 differentially alter MAVS70-dependent signaling, i.e., inhibiting IRF activation and IFN induction while weakly stimulating NF-κB activation? Based on our findings, we propose the following hypothetical model that summarizes the action of MAVS50 in specific inhibiting IFN induction (Fig 7). Upon stimulation such as activated RIG-I, MAVS70 forms large oligomers in the form of prion-like polymers or fibrils, resulting potent activation of both NF-κB and IRF transcription factors. By positioning TRAF-binding motifs at the very N-terminus, MAVS50 interacts more strongly with TRAF6, and likely TRAF2, than MAVS70. In doing so, MAVS50 efficiently sequesters TRAF6 from the prion-like MAVS70 polymers, attenuating the MAVS70-mediated signaling. When activated MAVS70 undergoes oligomerization, MAVS50 is induced to oligomerize via interacting with MAVS70. Given its high affinity for TRAF adaptor molecules, oligomerized MAVS50 is sufficient to induce NF-κB activation, but not IRF activation and IFN induction. Sum of both results is the specific inhibition of IFN induction and modest NF-κB activation by MAVS50. Then, how does MAVS70 activate both NF-κB and IRF, while MAVS50 activates only NF-κB. In SeV-infected or vGAT-expressing cells, MAVS70 migrated into the Triton X-100-insoluble fraction, whereas MAVS50 remained in the soluble fraction. In transfected 293T cells, MAVS50 forms smaller oligomers than MAVS70. These findings largely agree with the notion that higher order of MAVS70 oligomers form fibrils and precipitates out from Triton X-100-containing solution, whereas MAVS50 does not [8,31]. Together with TBK-1 that phosphorylates an IRF3-binding domain, MAVS70 fibrils provide a signaling platform that enables IRF3 activation and IFN induction [37]. Thus, it is conceivable that MAVS70 and MAVS50 form at least two types of oligomers that are of distinct sizes. The large oligomeric MAVS70 is capable of activating NF-κB and IRF, while the smaller MAVS50-containing oligomer only activates NF-κB. Perhaps, integration of MAVS50 into the MAVS70 larger oligomers shifts the signaling capacity of the complex from activating both NF-κB and IRF to that activating only NF-κB. This possibility remains to be formally tested in the future.

Compared to MAVS70, MAVS50 preserves all three TRAF-binding motifs, i.e., TRAF2- and TRAF6-binding motifs. Indeed, MAVS50 demonstrated preferential interaction with TRAF2 and TRAF6, while MAVS70 interacted with five TRAFs except TRAF4. This result agrees with the notion that the N-terminally exposed TRAF-binding motifs are better accessed and endow MAVS50 better interactions with TRAF2 and TRAF6. Intriguingly, mutations within the putative TRAF2-binding motif, but not those within the predicted TRAF6-binding motif, abolished MAVS50 interaction with both TRAF2 and TRAF6. Similarly, the MAVS50 M2,6 mutant was impaired to interact with TRAF3 and TRAF5. These results highlight the critical role of the very N-terminally exposed TRAF2-binding motif in interacting with more than one TRAF molecule, implying the degeneracy of the TRAF2-binding motifs in recruiting TRAF molecules and relaying signal transduction. It is important to note that, compared to MAVS50, these MAVS50 mutants were expressed well and interacted with MAVS70, suggesting that the defect in TRAF-binding is not due to overall protein misfolding. Alternatively, it is possible that TRAF2 recruited by the terminally positioned TRAF2-binding motif facilitates the interaction of MAVS50 with TRAF6 and other TRAFs [7,34]. This is supported by the observation that MAVS recruits multiple TRAFs with non-redundant roles in innate immune signaling [38] and TRAF molecules are prone to oligomerize [39]. The space between these two TRAF-binding motifs may not permit two TRAF molecules to dock on the N-terminus of MAVS50, but multiple TRAFs decorating on oligomerized MAVS50 are possible [32,33]. Nevertheless, by positioning the TRAF2-binding motif at the very N-terminus, MAVS50 effectively competes with MAVS70 for recruiting these key signaling molecules, resulting in attenuation of the MAVS70-mediated signal transduction.

The MAVS50 variant is generated from internal translation initiation, such that two TRAF2-binding motifs are exposed at the very N-terminus to facilitate protein interaction. Such a delicate mechanism, crafted by millions of years of evolution, symbolizes the importance of the tight regulation of MAVS-dependent innate immune signaling in response to viral infection. Notable regulatory action alike is exemplified by alternatively spliced variant of RIG-I [40] and TRIF [41], and a LGP2 allele likely due to gene duplication [42,43]. While alternative splicing has been extensively studied, the contribution of internal translation initiation in regulating fundamental biological processes such as immune response is not well understood. On the other hand, internal translation initiation is one of the mechanisms that viruses frequently deploy to maximize the coding capacity of their limited genomes [44,45] and that critical cellular genes avoid shutdown under stressed conditions [46,47]. It is probable that internal translation initiation of MAVS50 is activated to enhance its expression in virus-infected cells that the cap-dependent translation is suppressed. Expression of MAVS50 will alleviate and prevent an overacting antiviral immune response, thereby promoting cell survival. The roles of MAVS50 under this stressed condition remain to be determined.

## Materials and Methods

### Plasmids

Unless otherwise specified, all genes were cloned into pcDNA5/FRT/TO (Invitrogen) for transient expression, and pCDH-EF-puro-CMV-MCS (System Bioscience) for lentiviral expression. All cloned cDNAs were confirmed by DNA sequencing.

### Cell Lines and Viruses

HEK 293T (ATCC), HEK 293T-Rex cells (Invitrogen), BHK21 (ATCC), THP-1 (ATCC) were maintained in Dulbecco’s modified Eagle’s medium (DMEM) supplemented with 10% fetal bovine serum (FBS), penicillin (100 U/mL), and streptomycin (100 μg/mL). Human Jurkat (ATCC) T lymphoid cells were maintained in RPMI 1640 supplemented with 10% fetal bovine serum (FBS), penicillin (100 U/mL), and streptomycin (100 μg/mL). All cells were cultured at 37°C in an atmosphere of 5% CO_2_. VSV-GFP virus was amplified in BHK-21 cells. Viral titers were determined by a plaque assay using NIH3T3 monolayer.

### Antibodies

The following antibodies were used in this study: Anti-human MAVS^1-135^ (Santa Cruz Biotechnology), anti-human MAVS^150-250^ (Abcam), anti-human MAVS^150-200^ (Bethyl group), anti-β-actin (Abcam), anti-Flag (Sigma), anti-V5 (Bethyl Group), anti-HA (Covance). Antibodies against IKKβ and IKKγ were kindly provided by Dr. Ebrahim Zandi (University of Southern California).

### Luciferase Reporter Assay

Luciferase reporter assays were performed as previously described [48]. Briefly, HEK293T cells (1 x 10^5^ cells/well) were seeded in 24-well plates 16 hours prior to transfection. Cells were transfected with NF-ĸB or IFN-β reporter plasmid cocktail (including 50 ng of NF-ĸB or IFN-β promoter luciferase reporter plasmid and 100-ng of pGK-β-GAL plasmid) and an expression plasmid, by calcium phosphate transfection method. At 30 hours post-transfection, cell lysates were used to measure the firefly luciferase activity and β-galactosidase activity.

### Immunoprecipitation and Immunoblotting

Immunoprecipitation and immunoblotting were carried out as previously described [22,23,49]. Briefly, cells were harvested, rinsed with ice-cold PBS, and lysed with NP40 buffer (50 mM Tris-HCL [pH 7.4], 150 mM NaCl, 5 mM EDTA, 1% NP40) supplemented with protease inhibitor cocktail. Centrifuged cell lysates were then pre-cleared with Sepharose 4B beads, and subjected to precipitation with antibody-conjugated agarose (Sigma) at 4°C for 4–6 hours. Precipitated proteins were extensively washed with NP40 buffer and eluted with 1x SDS-PAGE loading buffer by boiling at 95°C for 5–10 minutes.

For immunoblotting analysis, whole cell lysates (WCL) or precipitated proteins were resolved by SDS-PAGE, and transferred to nitrocellulose membrane. Immunoblotting analysis was performed with indicated primary antibodies and proteins were visualized with IRDye800- or IRDye680-conjugated secondary antibodies (Licor) using an Odyssey infrared imaging system (Licor).

### Gel Filtration

Gel filtration was performed as previously described by Zandi et al [11]. Briefly, WCL or purified proteins were applied to superpose 6 or superdex 200 column (GE Bioscience) and subjected to gel filtration analysis with buffer B (1 mM EDTA, 0.5 mM EGTA, 150 mM NaCl, 20 mM Tris-HCl [pH 7.6], 0.5% Triton X-100, 20 mM NaF, 20 mM β-glycerolphosphate, 1 mM Na_3_VO_4_, 5 mM benzamidine, 2.5 mM metabisulphite). Elutions were collected in 0.5 ml fractions and were analyzed by immunoblotting.

### Quantitative Real-Time PCR (qRT-PCR)

qRT-PCR was performed as previously described [22,23]. Briefly, total RNA was extracted from HEK293T cells using TRIzol reagent (Invitrogen). To remove genomic DNA, total RNA was digested with RNase-free DNase I (New England Biolab). First-strand cDNA was synthesized from 1 μg total RNA, using reverse transcriptase (Invitrogen). The abundance of cytokine mRNA was assessed by qRT-PCR, using SYBR Green Master Mix (Applied Biosystems). Human β-Actin was used as an internal control.

### Enzyme-Linked Immunosorbent Assay (ELISA)

Commercial ELISA kits used in this study include: Human IFNβ (Thermo Scientific) and human CCL5 (R&D Systems). The supernatants from cultured cells were collected at the indicated time points after Sendai Virus infection. Cytokine levels in the supernatants were assessed according to manufacturer’s instruction.

### *In Vitro* Kinase Assay

HEK293T cells were transfected with the indicated plasmids. Cells were lysed and anti-IKKγ antibody was used to precipitate endogenous IKKα/β/γ complexes. The precipitated complexes were subjected to *in vitro* kinase assay as previously described [49]. The kinase reaction mixture consisted of GST-IĸBαNT as the substrate, [γ-^32^P]ATP, and precipitated kinase complex in 20 μl kinase buffer (25 nM HEPES [pH 7.5], 50 mM KCL, 2 mM MgCl_2_, 2 mM MnCl_2_, 1 mM DTT, 10 mM NaF, 20 mM β-glycerolphosphate, and 1 mM sodium orthovandate). The reaction mixture was incubated at room temperature for 40 minutes. Phosphoryation of IĸBα was analyzed by autoradiography.

### Lentivirus-Mediated Stable Cell Line Construction

Lentivirus production was performed as previously described [22,48]. Briefly, HEK293T cells were transfected with packaging plasmids (DR8.9 and VSV-G) and the pCDH lentiviral expression plasmids or shRNA plasmids. At 72 hours post-transfection, supernatant was harvested and, if necessary, concentrated by ultracentrifugation. HEK293T cells were then infected with lentivirus in the presence of polybrene (8 μg/ml). Cells were selected and maintained in complete media.

### Subcellular Fractionation

Cells were lysed and homogenized using hypotonic buffer solution (20 mM Tris-HCl, pH 7.4, 10 mM NaCl, 3mM MgCl_2_). The homogenates were centrifuged at 500xg for 5 minutes. The supernatant (S1) was centrifuged at 5000xg for 10 minutes to precipitate crude mitochondria. The crude mitochondria fraction (P5) was then lysed and analyzed by immunoblotting.

### Statistical Analysis

The statistical significance (*P*-value) was calculated using unpaired two-tailed Student’s *t* test. A *P*-value of <0.05 was considered statistically significant.

## Supporting Information

S1 Fig Innate immune signaling of MAVS50. (A and B) 293T cells were transfected with plasmids containing V5-tagged MAVS70 (A) or MAVS50 (B) and those containing Flag-tagged TRAFs. Whole cell lysates (WCLs) were precipitated with anti-Flag M2 agarose. Precipitated proteins and WCLs were analyzed by immunoblotting with indicated antibodies. (C) 293T cells were transfected with plasmids containing indicated genes. WCLs prepared in kinase lysis buffer were precipitated with anti-TBK-1 antibody and TBK-1 was subjected to in vitro kinase assay with GST-IRF3C as substrate. GST-IRF3C was analyzed by PhosphoImager and TBK-1 by immunoblotting. WCLs were analyzed by immunoblotting with antibodies against MAVS and GST (RIG-I-N). (D) Transfection was carried out as in (C) and WCLs were analyzed by native gel electrophoresis for IRF3 dimerization. WCLs were analyzed by immunoblotting with antibodies against β-actin, MAVS and GST (RIG-I-N).
(TIF)

S2 Fig MAVS50 expression and its effect on innate immune signaling in 293T cells. (A) MAVS knockdown 293T cells, “reconstituted” with control lentivirus (Vec) or lentivirus carrying wild-type MAVS (WT), MAVS70 (70) or MAVS50 (50), were infected with Sendai virus (100 HA unit/ml) for 8 hours. Total RNA was extracted, cDNA was prepared and analyzed by real-time PCR with primers specific for indicated genes. (B and C) MAVS knockdown 293T cells, “reconstituted” with various MAVS expression as described in (A), were incubated with mitotracker (B), fixed and stained with corresponding primary and secondary antibodies. For peroxisome staining, antibody against the 70 kDa peroxisome membrane protein (PMP70) was used (C). Cells were analyzed with a Nikon E800M microscope equipped with CCD camera. (D) 293 T-Rex/MAVS50-Flag cells were established with hygromycin selection (100 μg/ml) and induced with doxycycline at indicated concentration for 48 hours. Whole cell lysates were analyzed by immunoblotting with anti-Flag (MAVS50) and anti-β-actin antibodies.
(TIF)

S3 Fig Co-elution of MAVS70 and MAVS50 in 293T cells infected with virus. 293T cells were mock-infected, or infected with Sendai virus (SeV, 100 HAU/ml) (middle three panels) and murine gamma herpesvirus 68 (γHV68, MOI = 1) (bottom panel) for indicated time. WCLs in Triton x-100-containing buffer were analyzed by gel filtration with Superose 6 column and fractions were analyzed by immunoblotting with anti-MAVS antibody.
(TIF)

S4 Fig MAVS50 modulates the IRF-IFN signaling cascade. (A and B) 293T cells were transfected with an IFN-β reporter cocktail, plasmids containing IKKε (A) or IRF3-5D (B) and increasing amount of a plasmid containing MAVS50. At 30 hours post-transfection, whole cell lysates (WCLs) were analyzed by luciferase assay to determine the activation of the IFN-β promoter. (C) 293T cells were transfected with plasmids containing indicated genes. WCLs were precipitated with anti-V5 agarose (MAVS). Precipitated proteins and WCLs were analyzed by immunoblotting with indicated antibodies.
(TIF)

S5 Fig Characterization of TRAF2- and TRAF6-binding motifs of MAVS50. (A) Diagram of MAVS50 carrying N-terminally positioned TRAF-2 and TRAF6-binding motifs in relation to MAVS70. The key residues of the TRAF2- and TRAF6-binding motifs were mutated into alanines as indicated. (B and C) 293T cells were transfected with indicated plasmids. Whole cell lysates (WCLs) were precipitated with anti-Flag [TRAF3 (B) or TRAF5 (C)]. Precipitated proteins and WCLs were analyzed by immunoblotting with indicated antibodies. (D) 293T cells were transfected with plasmids containing indicated genes. Immunoprecipitation and immunoblotting were carried out as in (B).
(TIF)
