# Supplementary figures and images for: An Internally Translated MAVS Variant Exposes Its Amino-terminal TRAF-Binding Motifs to Deregulate Interferon Induction
Source: PLoS Pathog. 2015 Jul 29;11(7):e1005060. doi: 10.1371/journal.ppat.1005060 (PMC4519330; doi:10.1371/journal.ppat.1005060)

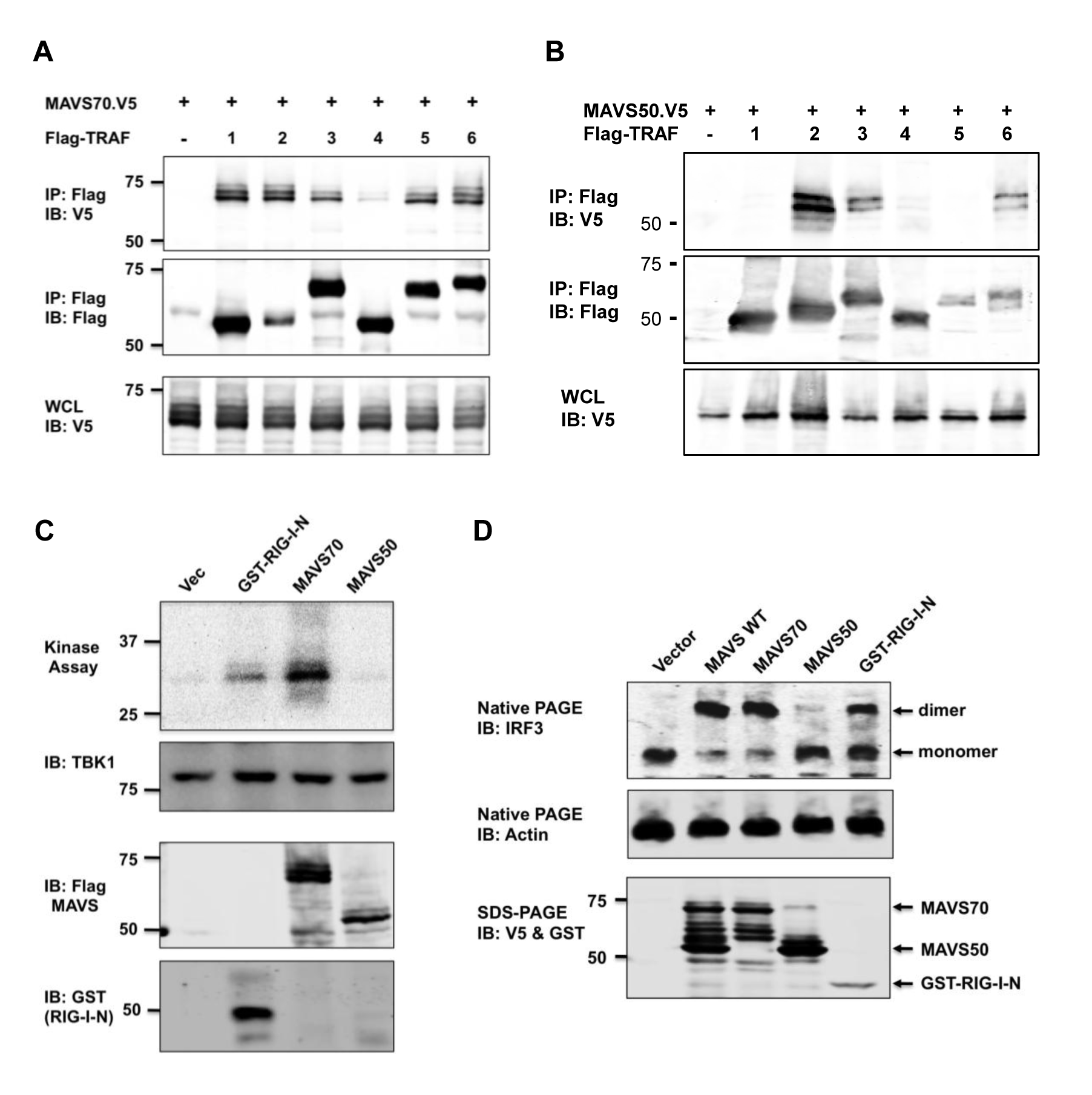

Supplement: S1 Fig — (A and B) 293T cells were transfected with plasmids containing V5-tagged MAVS70 (A) or MAVS50 (B) and those containing Flag-tagged TRAFs. Whole cell lysates (WCLs) were precipitated with anti-Flag M2 agarose. Precipitated proteins and WCLs were analyzed by immunoblotting with indicated antibodies. (C) 293T cells were transfected with plasmids containing indicated genes. WCLs prepared in kinase lysis buffer were precipitated with anti-TBK-1 antibody and TBK-1 was subjected to in vitro kinase assay with GST-IRF3C as substrate. GST-IRF3C was analyzed by PhosphoImager and TBK-1 by immunoblotting. WCLs were analyzed by immunoblotting with antibodies against MAVS and GST (RIG-I-N). (D) Transfection was carried out as in (C) and WCLs were analyzed by native gel electrophoresis for IRF3 dimerization. WCLs were analyzed by immunoblotting with antibodies against β-actin, MAVS and GST (RIG-I-N). (TIF) [file ppat.1005060.s001.tif]

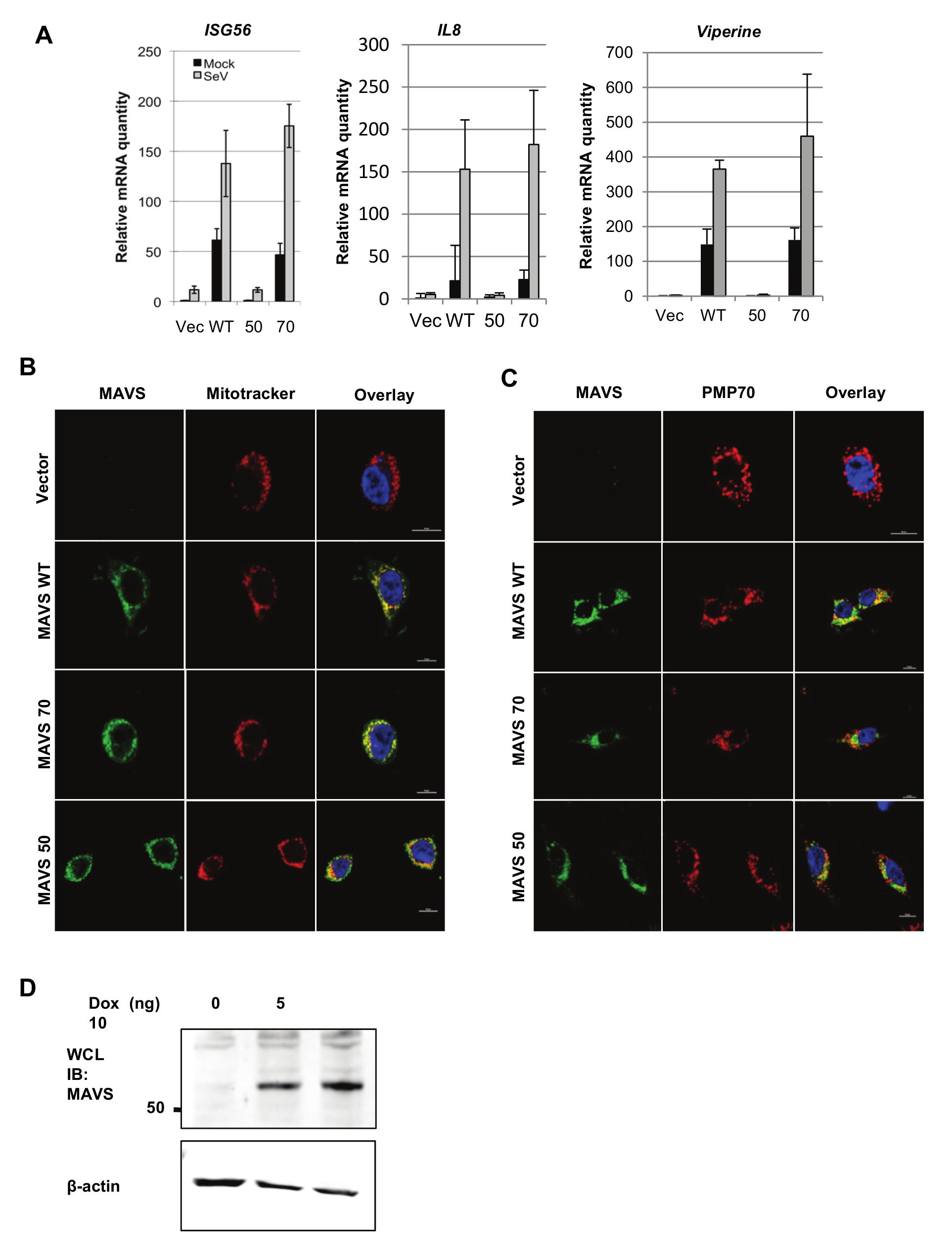

Supplement: S2 Fig — (A) MAVS knockdown 293T cells, “reconstituted” with control lentivirus (Vec) or lentivirus carrying wild-type MAVS (WT), MAVS70 (70) or MAVS50 (50), were infected with Sendai virus (100 HA unit/ml) for 8 hours. Total RNA was extracted, cDNA was prepared and analyzed by real-time PCR with primers specific for indicated genes. (B and C) MAVS knockdown 293T cells, “reconstituted” with various MAVS expression as described in (A), were incubated with mitotracker (B), fixed and stained with corresponding primary and secondary antibodies. For peroxisome staining, antibody against the 70 kDa peroxisome membrane protein (PMP70) was used (C). Cells were analyzed with a Nikon E800M microscope equipped with CCD camera. (D) 293 T-Rex/MAVS50-Flag cells were established with hygromycin selection (100 μg/ml) and induced with doxycycline at indicated concentration for 48 hours. Whole cell lysates were analyzed by immunoblotting with anti-Flag (MAVS50) and anti-β-actin antibodies. (TIF) [file ppat.1005060.s002.tif]

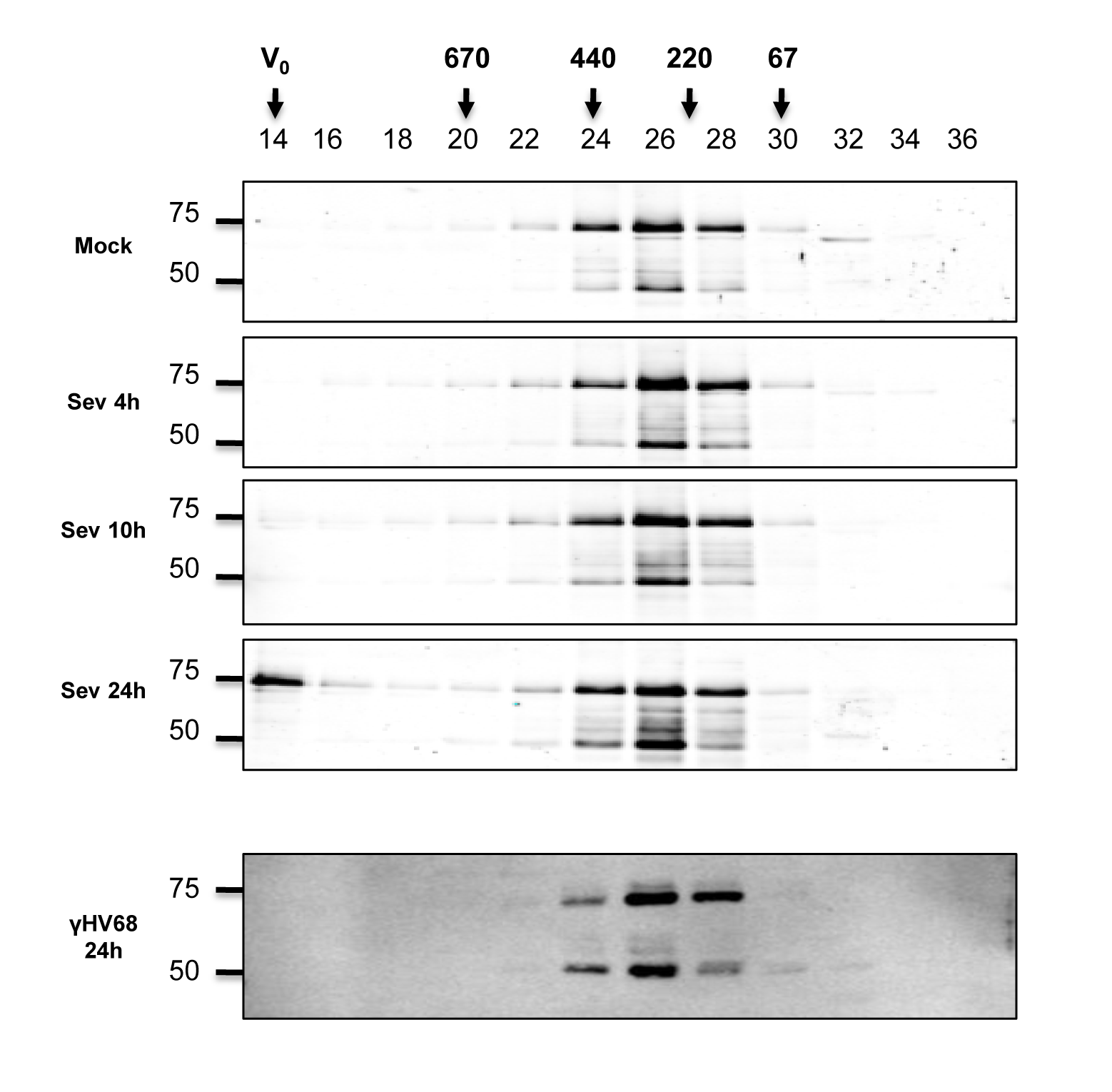

Supplement: S3 Fig — 293T cells were mock-infected, or infected with Sendai virus (SeV, 100 HAU/ml) (middle three panels) and murine gamma herpesvirus 68 (γHV68, MOI = 1) (bottom panel) for indicated time. WCLs in Triton x-100-containing buffer were analyzed by gel filtration with Superose 6 column and fractions were analyzed by immunoblotting with anti-MAVS antibody. (TIF) [file ppat.1005060.s003.tif]

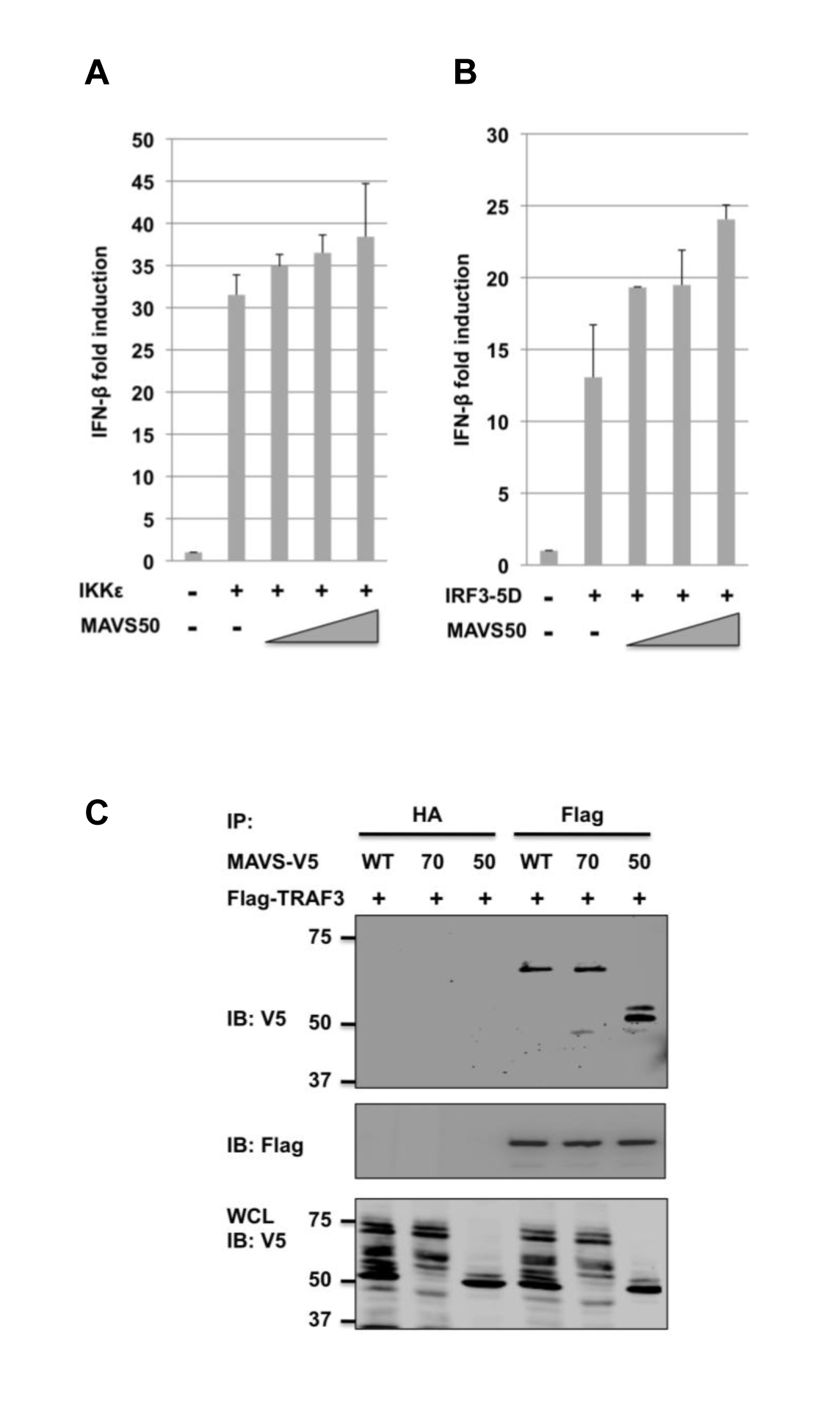

Supplement: S4 Fig — (A and B) 293T cells were transfected with an IFN-β reporter cocktail, plasmids containing IKKε (A) or IRF3-5D (B) and increasing amount of a plasmid containing MAVS50. At 30 hours post-transfection, whole cell lysates (WCLs) were analyzed by luciferase assay to determine the activation of the IFN-β promoter. (C) 293T cells were transfected with plasmids containing indicated genes. WCLs were precipitated with anti-V5 agarose (MAVS). Precipitated proteins and WCLs were analyzed by immunoblotting with indicated antibodies. (TIF) [file ppat.1005060.s004.tif]

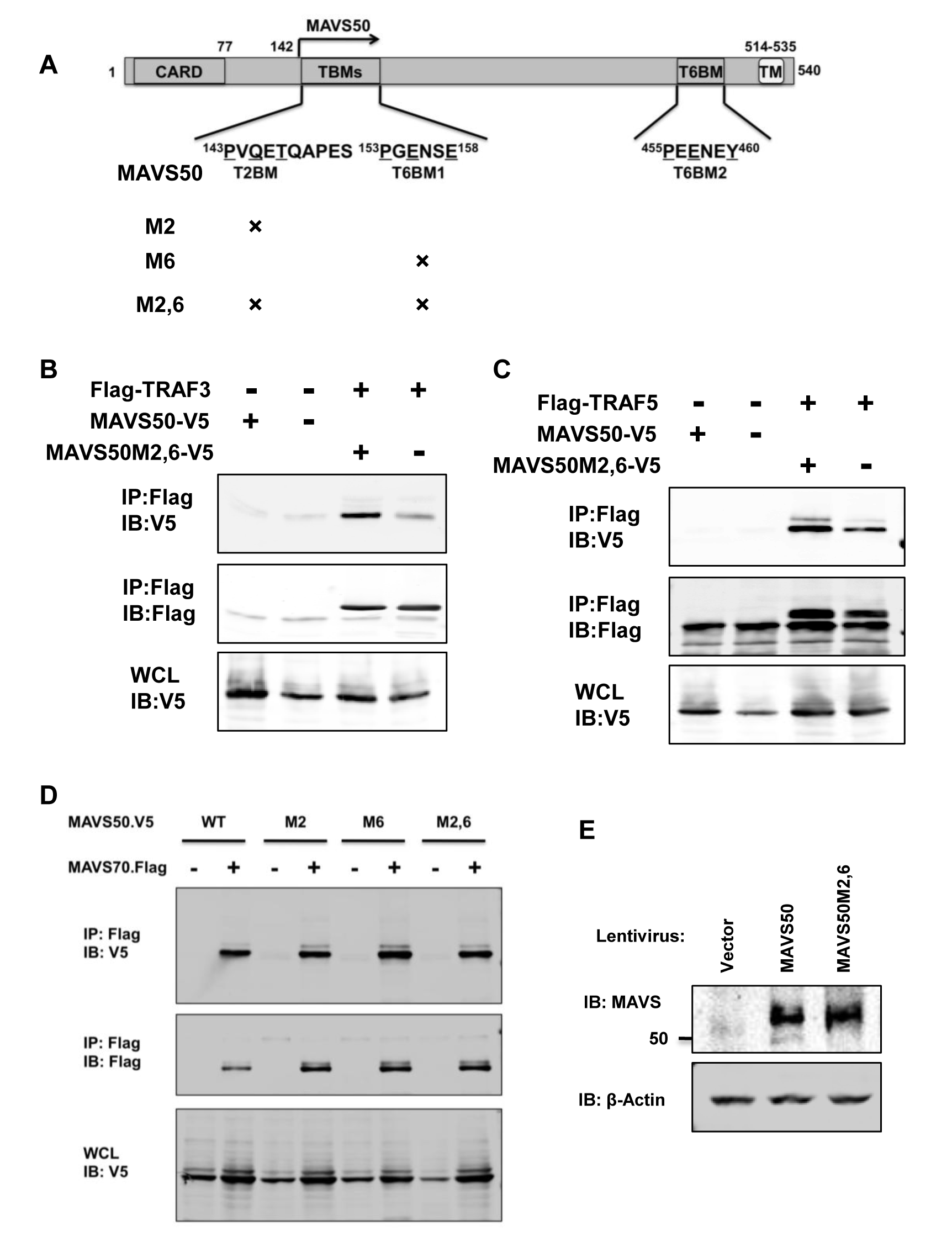

Supplement: S5 Fig — (A) Diagram of MAVS50 carrying N-terminally positioned TRAF-2 and TRAF6-binding motifs in relation to MAVS70. The key residues of the TRAF2- and TRAF6-binding motifs were mutated into alanines as indicated. (B and C) 293T cells were transfected with indicated plasmids. Whole cell lysates (WCLs) were precipitated with anti-Flag [TRAF3 (B) or TRAF5 (C)]. Precipitated proteins and WCLs were analyzed by immunoblotting with indicated antibodies. (D) 293T cells were transfected with plasmids containing indicated genes. Immunoprecipitation and immunoblotting were carried out as in (B). (TIF) [file ppat.1005060.s005.tif]
